# The rare nonsense mutation in p53 triggers alternative splicing to produce a protein capable of inducing apoptosis

**DOI:** 10.1371/journal.pone.0185126

**Published:** 2017-09-29

**Authors:** Evgeny M. Makarov, Tatyana A. Shtam, Roman A. Kovalev, Rimma A. Pantina, Elena Yu Varfolomeeva, Michael V. Filatov

**Affiliations:** 1 Division of Biosciences, College of Health and Life Sciences, Brunel University London, Uxbridge, United Kingdom; 2 National Research Centre "Kurchatov Institute" B.P. Konstantinov Petersburg Nuclear Physics Institute, Gatchina, Russia; 3 Peter the Great St Petersburg Polytechnic University, St Petersburg, Russia; 4 Petrov Institute of Oncology, St Petersburg, Russia; Virginia Commonwealth University, UNITED STATES

## Abstract

P53 protein is more frequently mutated in human tumours compared with the other proteins. While the majority of the p53 mutations, especially within its DNA-binding domain, lead to the loss of the wild-type function, there are accumulating data demonstrating that the p53 mutants gain tumour promoting activities; the latter triggers a revitalised interest in functional analysis of the p53 mutants. A systematic screening for p53 mutations in surgical materials from patients with glioma revealed a 378C>G mutation that creates a stop codon at the position of amino acid residue 126. The mutation eliminates the recognition site for the restriction endonuclease Sca I that allowed us to carry out RFLP analysis of DNA extracted from the clinical samples and suggests that this mutation is more frequent than is documented in the p53 databases. Both the ECV-304 and EJ cell lines, that probably originate from the bladder carcinoma T24 cell line, were confirmed to contain the homozygous 378C>G mutation but were shown to produce the p53 protein of expected full-length size detected by Western blotting. We provide evidence that the 378C>G mutation generates an alternative 3’ splice site (ss) which is more often used instead of the authentic upstream 3’ ss, driving the production of mRNA encoding the protein with the single amino acid deletion (p53ΔY126). Using endogenous expression, we demonstrated that the p53ΔY126 protein is nearly as active as the wild type protein in inducing the p21/Waf1 expression and apoptosis.

## Introduction

The p53 protein is a major player of numerous pathways that regulate cellular responses to stress. The p53 gene is the most commonly mutated gene in human cancers [1–3]. The p53 protein is considered to be a tumour suppressor because it blocks tumour development by triggering apoptosis or cellular senescence in response to oncogenic stress. Mutant p53 proteins are highly expressed in a variety of different cancer cells, and the majority of p53 mutations are missense mutations found in the DNA binding domain encompassing exons 5–8 of the gene [4,5]. Overall, p53 mutations are conventionally divided into two groups: those that alter the amino acid residues responsible for forming sequence-specific contacts with DNA, and those that change the p53 protein conformation which, in turn, affects the protein stability or disrupts interactions with its protein partners [6]. For many years, loss of p53 function through mutations, especially in the DNA binding domain which disrupts transcriptional regulation of target genes, was considered as the predominant contribution of p53 into oncogenesis [1]. This led to the hypothesis that restoration of wild-type p53 expression in tumour cells may eliminate malignant formations [7]. The idea was proven in a number of mouse model studies where it was shown that restoring the wild-type p53 activity, using different methodologies, led to tumour regression [8–10]. Another approach is a pharmacological reactivation of the mutant p53 which was successfully developed to restore the functionality of some of the p53 mutants using the small molecules, e.g., PRIMA-1 [11] and PK7088 [12]; the former is currently tested in the clinical trials [13].

New therapeutic strategies are likely to be developed in the future in view of emerging appreciation of novel oncogenic functions of the mutant p53 proteins. Instead of being focused solely on loss of wild-type functions in mutant p53, the research turned towards investigating a gain of tumour-promoting functions of mutant p53 [14,15]. Recently, gain-of-functions, including increased invasion, migration, and angiogenesis, were reported in a number of cell lines with different p53 mutations. Moreover, mouse models carrying the specific mutations of p53 often develop a wider repertoire of more aggressive tumours [15,16]. Several models explaining the mechanism of cellular action of the different p53 mutants were proposed and partially proven [16]. Most of these models rely on the ability of mutant p53 proteins to establish a new network of protein-protein interactions, or trigger conformational changes affecting either the p53-dependent transcriptional machinery or activating/downregulating expression of non-p53 target genes. However, more investigations are required to understand the molecular function of particular p53 mutants in certain cellular contexts.

Numerous cell lines with a variety of p53 mutations, originated from cancer patients, are in constant use in a great number of laboratories around the world; however, p53 mutational status and its effect on the cellular phenotype is still a controversial issue for many of the cell lines [17–19]. This can lead to erroneous interpretations of experimental results; therefore, a careful re-examination of p53 status of widely used cell lines is an important task.

This study investigates a rare C>G nucleotide substitution at position 378 of the p53 protein that creates a stop codon at the position of amino acid residue 126, and provides evidence that, in this mutant, premature termination of translation is avoided via a mechanism of alternative pre-mRNA splicing, generating the protein with the single amino acid deletion (p53ΔY126) that exhibits at least some functions of the wild-type protein.

## Materials and methods

### Cell lines and clinical samples

The HeLa human cervical carcinom*a*, K562 human chronic myeloid leukemia and ECV-304 transformed human endothelial cell lines were obtained from the Cell Culture Collection of the Institute of Cytology, Russian Academy of Sciences, St. Petersburg, Russia. The NB1-T human fibroblast cell line, generated from normal NB1 fibroblasts [20] by transfection with the hTERT cDNA [21] and EJ human bladder carcinoma cell line were from the Biosciences Cell Collection of Brunel University London, UK. The GL-V cell line was generated in our laboratory from surgical material of a patient with glioma provided by Polenov Institute of Neurosurgery, St. Petersburg, Russia. K562 cells were grown at 37°C with 5% CO_2_ in RPMI media and the other cell lines were cultured in DMEM; all supplemented with 10% foetal bovine serum and 1% penicillin-streptomycin. Formalin-fixed paraffin-embedded (FFPE) tumour tissue samples were received from Archives of Petrov Institute of Oncology, St Petersburg. The set of blood samples from cancer patients used in the RFLP analysis was obtained from Regional Hospital, Gatchina, Russia. Part of the research, including human materials acquired with informed consent, was approved by the Ethics Committee of Polenov Institute of Neurosurgery, St. Petersburg. The Institute is authorised to perform research involving human participants according to guidelines from the Ministry of Healthcare of the Russian Federation that are in agreement with the principles expressed in the Declaration of Helsinki.

### Plasmid constructions and cell transfections

The open reading frame (ORF) with 60 nucleotides of the 5′ untranslated region of the human wild type p53 gene, the first 125 amino acids (p53-aa125) and p53ΔY126 (amplified from the EJ cDNA), were cloned upstream and in-frame with EGFP into the vector pEGFP-N1. Transfections were performed 24 hours after seeding 5×10^4^ cells per well in 6-well tissue culture plates with 1 μg of the appropriate plasmids using Effectene Transfection Reagent (Qiagen) or X-tremeGENE HP DNA Transfection Reagent (Roche) according to manufacturer’s instructions. 48 hours after transfection, the cells were either examined for apoptosis by flow cytometry or collected for western blotting analysis. Attempts to generate stable cells were carried out by maintaining the transfected cells in media containing 400 μg/mL of antibiotic G418.

### Sub-cellular localisation of the p53-EGFP protein

NB1-T cells growing on glass coverslips in 6-well tissue culture plates were transfected with the aforementioned plasmids. 24 hours after transfection cells were fixed with 4% paraformaldehyde, counterstained with DAPI (5 μg/mL), and analysed by confocal fluorescent microscopy.

### RFLP analysis

DNA was extracted from cultured cells, FFPE and blood samples using PureLink Genomic DNA kit (ThermoFisher Sientific) according to the manufacturer’s instructions or by the routine Proteinase K-Phenol/Chloroform extraction method. DNA was quantified using a NanoDrop spectrophotometer and subjected to PCR amplification using using Phusion Hot Start DNA polymerase (ThermoFisher Scientific). The 453 base-pairs fragment from intron 4 to exon 6 of the p53 gene was produced using the In4.F forward primer—GCTGCCGTCTTCCAGTTGC and the Ex6.R reverse primer—CTCAGGCGGCTCATAGGGC, and then subjected to restriction enzyme digestion with Sca I followed by agarose gel analysis.

### The p53 cDNA analysis

Total RNA was extracted from ECV-304 and EJ cells using NucleoSpin RNA II kit (Macherey-Nagel) and converted into cDNA using random hexamer primers and Superscript III reverse transcriptase (Invitrogen); both procedures were carried out according to the manufacturer’s protocols. Alternatively, the cDNA was generated by reverse transcription using oligo(dT) primers. The 447 base-pair fragment of p53 ORF encompassing exon 4 to exon 6 was amplified using Phusion Hot Start DNA polymerase and the Ex4.F forward primer—GCACCAGCAGCTCCTACACC and the Ex6.R reverse primer. The PCR product was cloned into pJET2.1 vector and a number of clones were sequenced by GENEWIZ UK Ltd.

### Western blotting

For protein analysis of p53 and p21/Waf1 expression, whole cell extracts were prepared from 1–5 x10^6^ cells lysed in 30–50 μL of buffer containing 10 мМ Tris-HCl pH 7.4, 0.1% Triton X-100, 5 мМ PMSF, 5 мМ MgCl_2_, 5 u/mL DNAse I, 20 мМ β-mercaptoethanol, followed by sonication. 20 μg of total protein, as estimated by Bradford assay (Pierce), were resolved by 10% SDS–PAGE and transferred to a PVDF membrane. These were hybridised with either mouse monoclonal p53 antibody (clone DO-1, Sigma) at a 1:1,000 dilution, or p21/Waf1 (clone CP74, Millipore) at a 1:1,000 dilution followed by treatment with the peroxidase-labeled anti-mouse IgG (whole molecule) antibody (Sigma) at a 1:10,000 dilution. GAPDH (glyceraldehyde-3-phosphate dehydrogenase) was utilised as an endogenous control for equal loading using the monoclonal antibody ZG003 (ThermoFisher Scientific) at a 1:10,000 dilution. Antibody binding was detected by enhanced chemoluminescence (Pierce). Precision Plus Protein Dual Color standards (Bio-Rad) were used to estimate the molecular weight of the detected proteins.

### Cell viability assay

The assay was carried out according to Franken *et al*., 2006 [22]. Briefly, 5×10^3^ GL-V cells were seeded per well into 24-well tissue culture plates and were transfected after 24 hours with 0.4 μg of the corresponding plasmids using Effectene Transfection Reagent (Qiagen). The next day, G418 (Geneticin) was added to the medium to a final concentration of 400 μg/mL to select cells that had acquired neomycin resistance from the plasmid. These were cultivated for 2 weeks in the presence of antibiotic. Cells were fixed with glutaraldehyde and stained using crystal violet (0.1% in 20% methanol).

### Flow cytometry

The flow cytometric analysis of propidium iodide stained cells was employed to assess cell apoptosis as described by Belloc *et al*., 1994 [23]. Briefly, 48 hour after transfection, 1 mL of the K562 cell suspension containing 10^5^ cells was incubated with 100 μL of propidium iodide (50 μg/mL) for 4 minutes, and were analysed on a Beckman Coulter Cell Lab Quanta SC Flow Cytometer. The cells emitting green fluorescence (generated from the plasmid) were taken into consideration and compared with the number of cells emitting red fluorescence due to propidium iodide staining. Results of three individual transfections with each of the plasmids were used to calculate the percentage of apoptotic cells.

## Results

**Fig 1:**
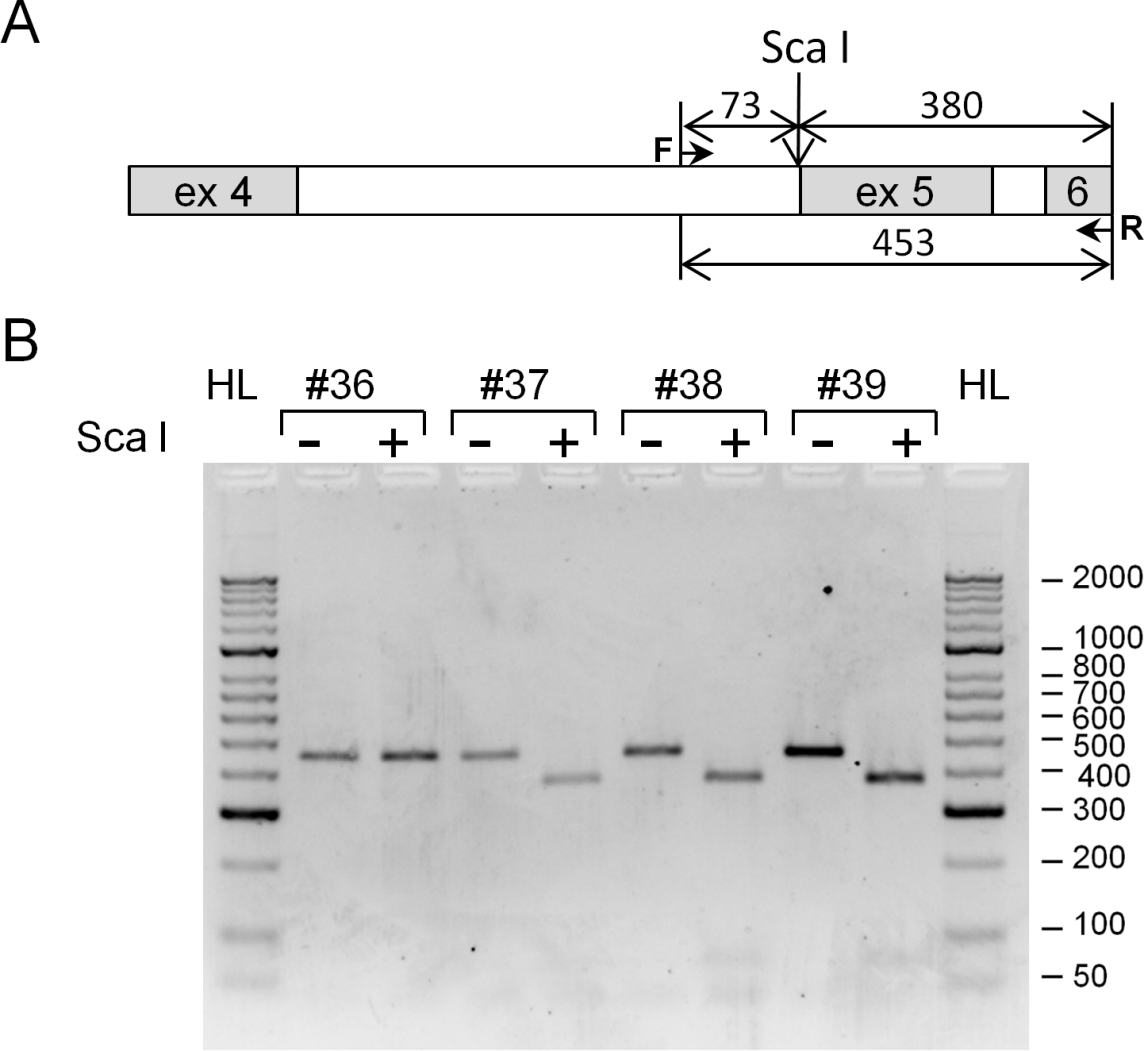
RFLP analysis of genomic DNA to detect the 378C>G mutation in the p53 gene. DNA, extracted from the four formalin-fixed paraffin-embedded tissue samples of colorectal cancer, was amplified using the primers annealing to intron 4 (forward) and exon 6 (reverse) and the PCR products were subjected to the Sca I restriction enzyme digestion. (A) Schematic presentation of amplified and the Sca I digested fragments on the p53 gene. Numbers indicate the sizes of DNA fragments in base pairs (bp). The band of 453 bp after the restriction enzyme digestion indicates the absence of Sca I site which is a characteristic of the 378C>G mutation. The band of 380 bp is a product of the Sca I restriction enzyme digestion and is synonymous to the wild type p53 gene. (B) Agarose gel electrophoresis of the amplified DNA fragments before (-) and after (+) the restriction enzyme digestion. Molecular weight markers from HyperLadder II (Bioline) are shown in bp on the right.

Mutation analysis of p53 via exon sequencing is commonly used in clinical practice for assessment of p53 functionality in surgically removed tumours. Through a joint programme with Polenov Institute of Neurosurgery, St Petersburg, Russia, we identified a C to G nucleotide substitution at position 378 of the p53 open reading (378C>G mutation) in one DNA sample extracted from surgical material of a patient with glioma. This mutation is considered to be rare according to IARC (International Agency for Research on Cancer, R18, April 2016 is the latest) p53 database and has not been reported previously in gliomas [4]. We realised that the 378C>G mutation eliminates the recognition site (AGT/ACT) for the Sca I restriction endonuclease allowing a simple RFLP (Restriction Fragment Length Polymorphism) analysis of genomic DNA using the Sca I restriction enzyme digestion. We employed this analysis to the samples available to us from Petrov Institute of Oncology, St Petersburg, and Regional Hospital, Gatchina, Russia. This included DNA extracted from 11 formalin-fixed, paraffin-embedded (FFPE) tissue sections of colorectal cancer, 13 FFPE of gastric cancer, and 18 samples of DNA extracted from lymphocytes of patients with the different types of cancer. Fig 1 shows an example of RFLP analysis. We have found one DNA sample (#36 in Fig 1) originated from FFPE tissue sections of colorectal cancer that contains the same 378C>G mutation (also verified by sequencing) out of 42 tumor samples available to us. This indicates that this mutation may occur more frequently in cancer patients compared to the p53 database where it was reported in seven different cell lines [4]. The Sca I restriction digestion analysis could be easily employed to accumulate statistical data on frequency of this mutation.

To investigate the effect of the 378C>G mutation at the cellular level, we performed *in silico* analysis of the p53 and the cell line databases and found three cell lines containing the same mutation: ECV-304—initially described as a spontaneously transformed cell line derived from a Japanese human umbilical vein endothelial cell culture; EJ and T24 –both are urinary bladder carcinoma. These cell lines are subject to controversy as they are suspected of being cross-contaminated, and are currently believed to be originating (if not identical) from the T24 cell line (see Discussion). However, since both ECV-304 and EJ cells were available to us, we decided to investigate the p53 status in these cell lines. Moreover, there were contradictory data on p53 mutations in EJ cells varying from no mutations [24] to point mutations at the different nucleotides [25,26]. We carried out sequencing analysis of genomic DNA and cDNA and confirmed the presence of the 378C>G mutation in both ECV-304 and EJ cells from our collections. We have also revealed that both ECV-304 and EJ cells are homozygous in respect to the 378C>G mutation as no Sca I digestion was detected by RFLP analysis of their genomic DNA. No mutation at position 490A>G (K164E), reported by Rieger *et al*., 1995 [25], was detected either in ECV-304 or EJ cell line, and we did not find any other mutations in addition to the 378C>G mutation in the p53 cDNA from our cell lines.

**Fig 2:**
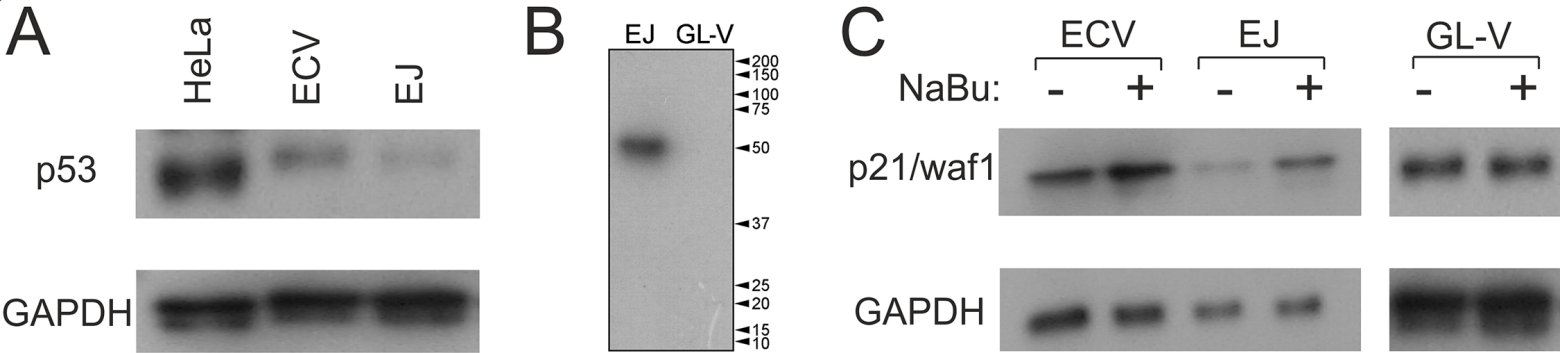
Analysis of p53 expression in ECV-304 and EJ cells. (A) Western blotting analysis of p53 protein in HeLa, GL-V, ECV-304 and EJ cells. (B) The full-length Western blot of EJ and GL-V cells probed with the p53 antibody. The molecular weight protein markers are indicated on the right. (C) Immunodetection of p21/Waf1 protein in ECV-304, EJ and GL-V cells before (-) and after exposure to 10 mM of sodium butyrate (NaBu) for 24 h (+). Immunodetection of GAPDH protein is used as an endogenous control.

A C>G nucleotide substitution at position 378 of the p53 open reading frame creates the stop codon (TAG) instead of tyrosine at amino acid position 126 (Y126 –TAC). Since this is a homozygous mutation, all the mRNAs encoding the p53 protein should contain the stop codon which could trigger nonsense mediated decay (NMD) of the p53 mRNA or, at best, lead to production of truncated protein of 125 amino acids exclusively. However, Western blotting analysis revealed the presence of p53 protein of expected full-length size in both ECV-304 and EJ cells (Fig 2A). Moreover, this is the only protein detected by the DO-1 monoclonal p53 antibody that recognises the N-terminal epitope mapped between amino acid residues 11–25 of human p53 protein (Fig 2B). As a negative control, the GL-V cell line generated in our laboratory (St Petersburg, Russia) from the surgical material of the patient with glioma, was used because it contains an extended deletion of the p53 gene encompassing almost the entire open reading frame (S1 File, Supporting Information). p53 dependent cell cycle arrest mediates the transactivation of the p21/Waf1 gene, which encodes the inhibitor of cyclin-dependent kinases [27]. Sodium butyrate (NaBu), a histone deacetylase inhibitor, induces p21/Waf1 expression [28], hence we investigated the effect of NaBu on ECV-304 and EJ cells. Indeed, p21/Waf1 protein level was increased in response to NaBu treatment in both ECV-304 and EJ cells, whereas no the p21/Waf1 induction was observed in GL-V cells where the p53 gene is deleted (Fig 2C).

**Fig 3:**
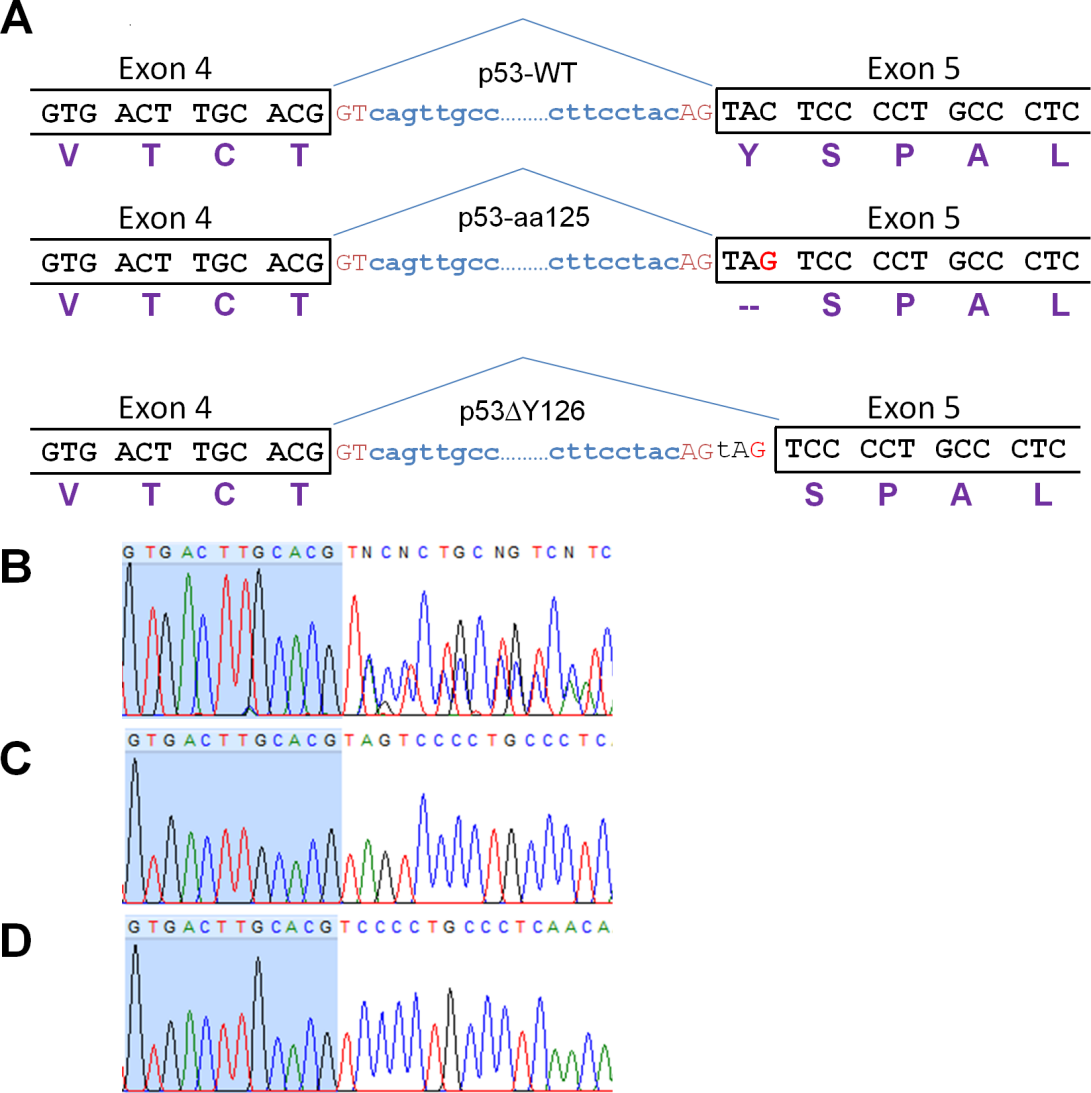
Sequence analysis of the 378C>G mutation in p53 protein. (A) Schematic representation of the Exon 4-intron-Exon 5 boundaries demonstrating how the G for C substitution creates an alternative 3’splice site which, if used, eliminates the stop codon from the mutated mRNA. The three proteins are designated here as p53-WT, p53-aa125, p53ΔY126. (B) Sequence analysis of exon 4-exon 5 junction in the PCR product amplified from cDNA of EJ cells. (C, D) The representative chromatograms of the sequenced clones corresponding to p53-aa125 and p53ΔY126, respectively. The nucleotides of exon 4 are highlighted in blue. The chromatogram was generated using FinchTV software.

It is evident that a discrepancy exists as all of the mRNAs encoding the p53 protein in ECV-304 and EJ cells can only drive the production of truncated protein of 125 amino acids, yet the presence of a full-length protein with the Western blotting is observed. Moreover, this p53 protein is functionally active if it is involved in the NaBu-dependent induction of p21/Waf1 expression. One possibility that may account for this occurrence is a stop codon readthrough. However, the premature termination codons (PTC) are readthrough by the ribosomes with such a low frequency that this phenomenon can hardly explain the amount of p53 detected on the blots (see Discussion). The other possibility is that the 378C>G mutation generates an alternative 3’ splice site (ss) which, if efficiently used instead of the authentic upstream 3’ ss, would lead to the production of mRNA encoding the protein with single amino acid deletion (Y126) (Fig 3A). The p53ΔY126 protein is unlikely to be distinguished from a full-length protein by their migration on SDS-PAGE, hence the signal on the Western blots of ECV-304 and EJ proteins (Fig 2A) is the p53ΔY126 protein. To investigate this hypothesis, we amplified the DNA fragments from exon 4 to the end of the exon 6 from cDNA generated from total RNA extracted from ECV-304 and EJ cells, and sequenced the PCR product. Fig 3B shows the mixed sequence starting after the first nucleotide of exon 5 (position 376 in p53 ORF) which would correspond to the two splice isoforms: ACG/TAGTCC and ACG/TCC, the latter represents the deletion of the mutated Y126 (TAG>TAC). To confirm the sequencing and estimate the frequency of the alternative splicing event, we cloned the PCR product into pJET2.1 vector, and sequenced a number of clones. Analysis revealed that 10 out of 20 sequenced clones from the ECV-304 cDNA and 10 out 14 sequenced clones from the EJ cDNA contain the deletion of three nucleotides encoding Y126 (Fig 3D). The rest of the clones contain TAG instead of Y126 corroborating the genomic sequencing (Fig 3C). Thus, the nonsense mutation at the beginning of exon 5 indeed activates the alternative 3’ss, allowing the stop codon to be skipped and p53 protein being synthesised with the single amino acid deletion in more than 50% of splicing events. The presence of mRNAs containing the stop codon contradicts to the fact these RNAs meet the requirements for nonsense-mediated decay (NMD) and are supposed to be degraded. Moreover, the truncated protein of 125 amino acids was not detected by Western blotting (Fig 2B). To reveal whether the RNAs contain the poly(A) tail or not, we carried out reverse transcription of total RNA from EJ cells using oligo(dT); amplified the fragment encompassing mutation; cloned the PCR products and sequenced. We found that 3 out of 13 sequenced clones contain the stop codon that is similar (4 out of 14) to the EJ cDNA generated using random hexamers. The result indicates that a number of mRNAs containing the stop codon may escape NMD, and therefore, the truncated protein of 125 amino acids (p53-aa125) can be potentially synthesised.

**Fig 4:**
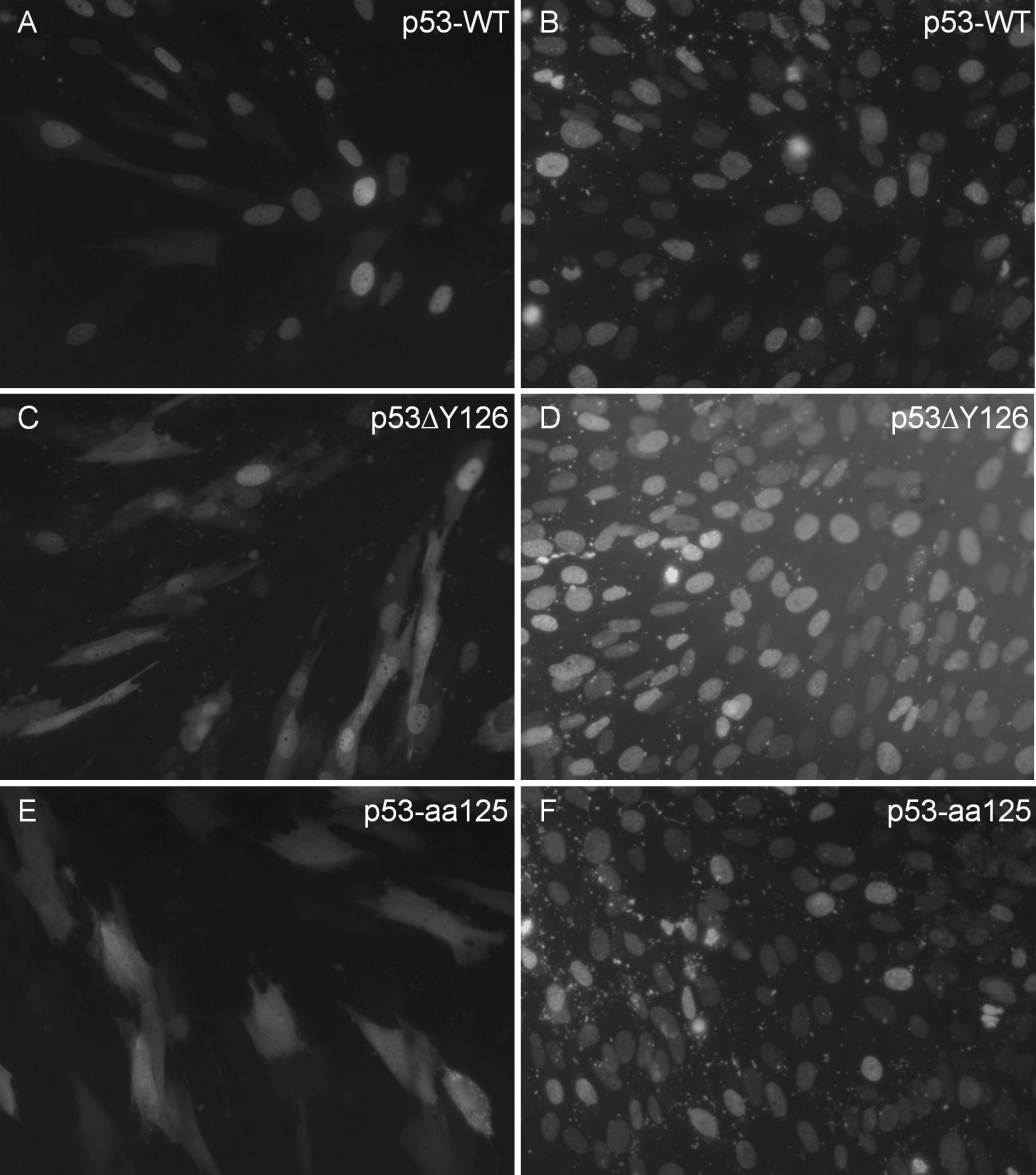
**p53-EGFP expression in NB1-T cells 24 hours after transfection with plasmids driving the expression of the wild type p53 (A, B), p53**Δ**Y126 (C, D), p53-aa125 (E, F).** Panels A, C, E show green fluorescence originated from the EGFP; panels B, D, F–blue fluorescence from counterstaining with DAPI.

Next, we investigate whether the p53ΔY126 protein has functional properties similar to the wild type protein or not. Since the mRNAs encoding the p53-aa125 were detected in our RT-PCR analysis, we decided to include also the truncated protein in our functional assays. The genetic constructs for eukaryotic expression of p53ΔY126, p53-aa125 and the wild type p53 as the N-terminal fusions to the enhanced green fluorescent protein were generated using the pEGFP-N1 vector, and first, were transiently transfected into immortalised human fibroblasts. Both the wild type p53 and p53ΔY126 were predominantly localised in the nuclei (Fig 4A and 4C, respectively) in 24 hours after transfection, consistent with previous observations in which the p53wt-EGFP fusion protein was detected in the nuclei [29]; whereas the p53-aa125 protein was distributed through the entire cell (Fig 4E); a similar staining was produced with a parental vector alone.

**Fig 5:**
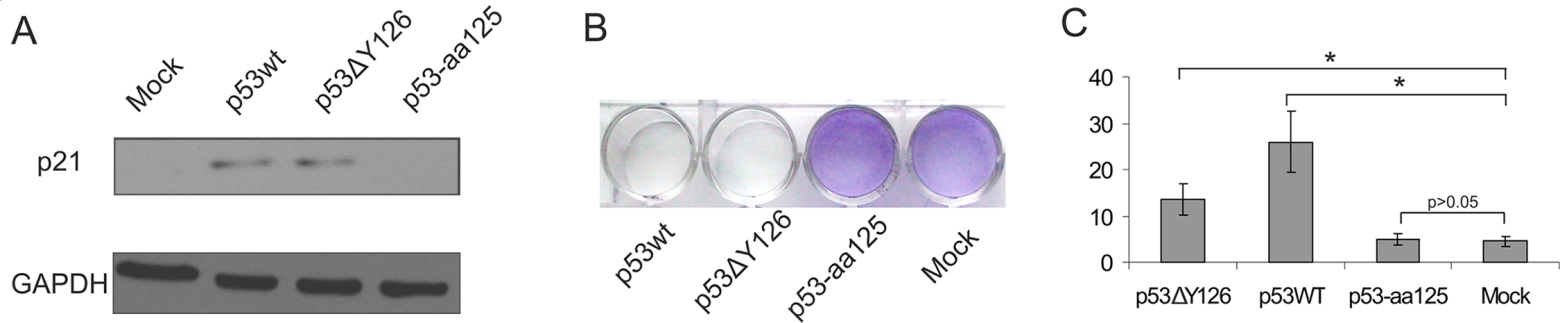
Functional properties of the p53ΔY126 and p53-aa125 proteins compared to the wild type protein. (A) Induction of the p21/Waf1 expression by transient transfection of K562 cells with plasmids expressing different variants of p53 proteins. Mock corresponds to transfection with the vector pEGFP-N1. Cells were collected 24 hours after transfection, and the total cell extracts were analysed by Western blotting with the p21/Waf1 antibody and the GAPDH antibody used as an endogenous control. (B) Cell viability assay for the GL-V cells transfected with the corresponding plasmids. (C) Flow cytometry analysis of the p53-induced apoptosis in the K562 cells transfected with the corresponding plasmids. The paired t-Test was used for statistical analysis of the mock transfected cells and the cells transfected with p53-aa125 or p53ΔY126 or p53wt using data from at least three independent experiments. Asterisk (*) represents the statistical significant difference with P-value <0.05.

The other experiments were carried out using the cell lines with p53 null genotype to avoid competition between endogenously and exogenously expressed p53 proteins. When the plasmids were transfected into K562 cells, all the p53-EGFP fusion proteins were expressed as confirmed by Western blotting (S2 File, Supporting Information). After transfection of K562 cells, p53ΔY126 induced the p21/Waf1 expression as well as the wild type p53, whereas p53-aa125 failed to do it as well as the carrier vector (Fig 5A). These results indicate that the p53ΔY126 protein is able to regulate one of the essential p53 target genes. The truncated p53-aa125 protein does not contain the DNA binding domain, and therefore, its inability to activate down-stream genes was not surprising.

One of the most important p53 functions is its ability to induce apoptotic cell death. To assess this activity of the p53ΔY126 protein, we first performed a cell viability assay using GL-V cells [22]. Fig 5B shows that the cells transfected with the plasmids driving the expression of either the wild type or the p53ΔY126 protein eventually died when were cultivated in the presence of antibiotic G418, whereas the cells expressing the p53-aa125 protein survived. Consistent with this result, we were unable to select stably transfected cell lines expressing the wild-type p53-EGFP fusion or p53ΔY126-EGFP following successful transient transfections of the cells carrying the wild-type p53 gene (see Fig 3 as an example) as all the clones initially emitting fluorescence were dying upon antibiotic selection. Finally, using flow cytometry we measured the induction of apoptosis in K562 cells at 48 h after transfection [23]. If p53-aa125 was inactive, the expression of both the wild-type and p53ΔY126 proteins induced apoptosis albeit the number of apoptotic cells transfected with the mutant was approximately 2-fold lower compared with the wild type (Fig 5C). Overall, our results demonstrate that the p53ΔY126 protein is nearly as active as the wild type protein as far as the p21/Waf1 induction and the p53-induced apoptosis are concerned, whereas the 125aa truncated protein is silent in this respect.

## Discussion

P53 is commonly recognized as the most important tumour suppressor protein due to its role in inducing cell cycle arrest or programmed cell death; thus preventing uncontrolled cell divisions or propagation of the cells with damaged genomes. Most of its functions were discovered from studies using cells carrying mutations in the p53 gene. Historically, p53 mutations leading to the loss of the wild-type function were thought to provide the main contribution of p53 in developing cancer. However, the emerging role for p53 mutants in tumour formation and progression via a gain-of-function mechanism has already generated increased interest in studying the impact of the specific p53 mutants at molecular, cellular and physiological levels.

This study has focused on a C to G nucleotide substitution at position 378 of the p53 protein, which we identified in two clinical samples derived from brain and colorectal cancer. We screened the p53 and cell lines databases and found the cell lines carrying 378C>G mutation; two of them–EJ and ECV-304 were available to us. These cell lines are not our preferred choice, but it was inevitable one as the only way to study this mutation at the cellular level. EJ and ECV-304 were widely used cell lines since early 1980s and 1990s, respectively [30–32] but were included into the list of suspicious cell lines that are likely to be cross-contaminated or misidentified many years ago. Indeed, the early short tandem repeat (STR) profiling revealed that ECV-304, EJ and T24 are characterised by the identical STR pattern [17]. The most recent detailed annotations of the cell line inventory suggest these three cell lines are likely to be the same and now consider the ECV-304 and the EJ cell lines as contaminants, and the human T24 bladder carcinoma cell line as a parental or authentic one [19,33]. Despite of this, all the three cell lines are still currently used in many laboratories around the world. In addition, there are contradictory data on the p53 mutational status in EJ cells varying from no mutations [24] to point mutations at the different nucleotides [25,26] as well as some controversy about positive immunological staining of p53 protein in the cells and negative detection on Western blots.

Sequencing analysis revealed that our ECV-304 and EJ cells contain only one single nucleotide mutation - 378C>G. This is present on both alleles of the p53 gene and creates a stop codon (TAG) instead of tyrosine at amino acid position 126 (Y126 –TAC). Unexpectedly, a band corresponding to the full-length p53 protein size was detected on the immunoblots whereas the truncated 125-aa protein was not. This could only be explained at the post-transcriptional level. There is a natural phenomenon, a stop codon readthrough, that occurs at authentic termination codons, albeit at a very low frequency (10^−4^) [34]. The premature termination codons (PTC) are readthrough by the ribosomes much more often with a help of the close-loop mRNA structure formed via interactions between the proteins bound to the 5’ end and to the poly(A) tail at the 3’ end of mRNA; however, frequencies of termination suppression at PTCs are still below 1% [35]. Thus, a stop codon readthrough can’t solely explain the presence of full-length p53 protein in EJ and ECV-304 cells.

The other possibility is an alternative pre-mRNA splicing. Indeed, we provide here the evidences that the nonsense mutation at the beginning of exon 5 generates the alternative 3’ss which is used in more than 50% splicing events. This allows the splicing machinery to skip a stop codon and synthesise the p53 protein with a single amino acid deletion (p53ΔY126). The 378C>G mutation creates the NAGNAG motif that contains two 3′ ss in tandem. Hiller et al., 2004 [36] estimated that the NAGNAG motif is present in 30% of human genes and suggested that it may play a functional role in about 5% of the genes. Using an EST-derived alternative splicing database, Dou at al., 2006 [37] provided evidences that the NAGNAG motif is indeed subjected to alternative splicing in about 50% cases. Nevertheless, analysis of different nucleotides at position N of the NAGNAG consensus sequence [38] does not support preferential use of the downstream 3’ ss in the context of the mutant 378C>G. At the same time, our results cast some support in favour of a general mechanism that triggers the alternative splicing events to avoid a PTC [39].

Interestingly, Liu and Bodmer, 2006 [40] reported the presence of the 378C>G mutation in the colorectal cancer cell line CC20 as well as the detection of p53 protein by antibody DO-1 (the same antibody used in this study); however, it is not clear whether the truncated protein of 125 aa or a full-length protein was detected there. It is also possible that the tongue squamous cell carcinoma BICR 56 cell line also carries the 378C>G mutation [41]. The authors reported the deletion of 21 nucleotides in the p53 cDNA prepared from BICR 56 cells that corresponds to an in-frame deletion of seven amino acid residues 126–132, and could be triggered by stop codon mutation at the genomic level. If proven, it would be an interesting example of how one nonsense mutation can activate two, probably competing, alternative splice sites, but both would save an open reading frame.

RT-PCR analysis revealed that at least 23% of all mRNAs encoded by the p53 gene in EJ and ECV-304 cells contain the stop codon, despite meeting the requirements for degradation via NMD. Mammalian NMD is triggered when a ribosome stalls at the PTC, which is located over 55 nt upstream of an exon-exon junction, and forms a network of protein-protein interactions with the exon-junction complex which is deposited during splicing on mRNA 20–25 nt upstream of exon-exon junction [42]. Even if the importance of NMD is underscored by the fact that about 30% of mutations linked to human diseases generate PTCs [43], we are still far from elucidation of the NMD pathway as there are experimental data showing that not every PTC triggers NMD and that an abundance of some authentic mRNAs is also regulated via NMD.

If the p53 mRNAs containing the stop codon escape NMD in EJ and ECV cells, the truncated protein of 125 amino acids (p53-aa125) can be synthesised; however, we were unable to detect this protein using the p53 antibody recognising the N-terminal epitope. This indicates that either the truncated protein is rapidly degraded or our RT-PCR analysis partially detected the RNAs which have not been released from the nucleus yet. Nevertheless, if the truncated protein is produced in the cells, it is very unlikely that it exhibits dominant-negative properties as we didn’t find any activity of p53-aa125 in our assays. In contrast, the p53ΔY126 protein, transiently produced in the cells lacking the p53 gene, induces the expression of one of the important target genes in the p53 pathway—p21/Waf1. The p53ΔY126 protein also causes cell death with efficiency comparable to the wild-type protein, despite the absent tyrosine is a highly conserved residue (S3 File, Supporting Information). Also, consistent with numerous data that the expression of p53 induces apoptosis [44–46], we have failed to generate a stable cell line following the successful transient expression of either the wild type p53 or p53ΔY126 protein fused to EGFP.

Our work highlights the lack of extensive experimental data on the p53 mutations that can potentially trigger alternative pre-mRNA splicing responsible for the production of p53 proteins which may exhibit new properties affecting the cellular functions. Blocking the production of such a protein may provide new therapeutic targets; amongst them could be the components of splicing machinery [47].

## Supporting information

S1 File Characterisation of the p53 status in a panel of human cell line. (PDF)

S2 File The p53 expression in the K562 cells transfected with plasmids encoding the p53-aa125-EGFP, p53ΔY126-EGFP and p53wt-EGFP proteins. (PDF)

S3 File Tyrosine at amino acid position 126 of p53 protein is a conserved residue among different species. (PDF)
