# Supplementary material for: The rare nonsense mutation in p53 triggers alternative splicing to produce a protein capable of inducing apoptosis
Source: PLoS One. 2017 Sep 29;12(9):e0185126. doi: 10.1371/journal.pone.0185126 (PMC5621691; doi:10.1371/journal.pone.0185126)
Supplement: S1 File — (PDF) [file pone.0185126.s001.pdf]

Supporting Information – S1 File

Analysis of the p53 genomic DNA and cDNA in ECV-304, EJ and GL-V cell lines

The region encompassing exon 4-5-6 of p53 was amplified from cDNA (Fig S1-1A) or genomic DNA (Fig S1-1B) and subjected to agarose gel electrophoresis analysis. The LMNA, ACTN4 and hTERT genes were used as endogenous controls.

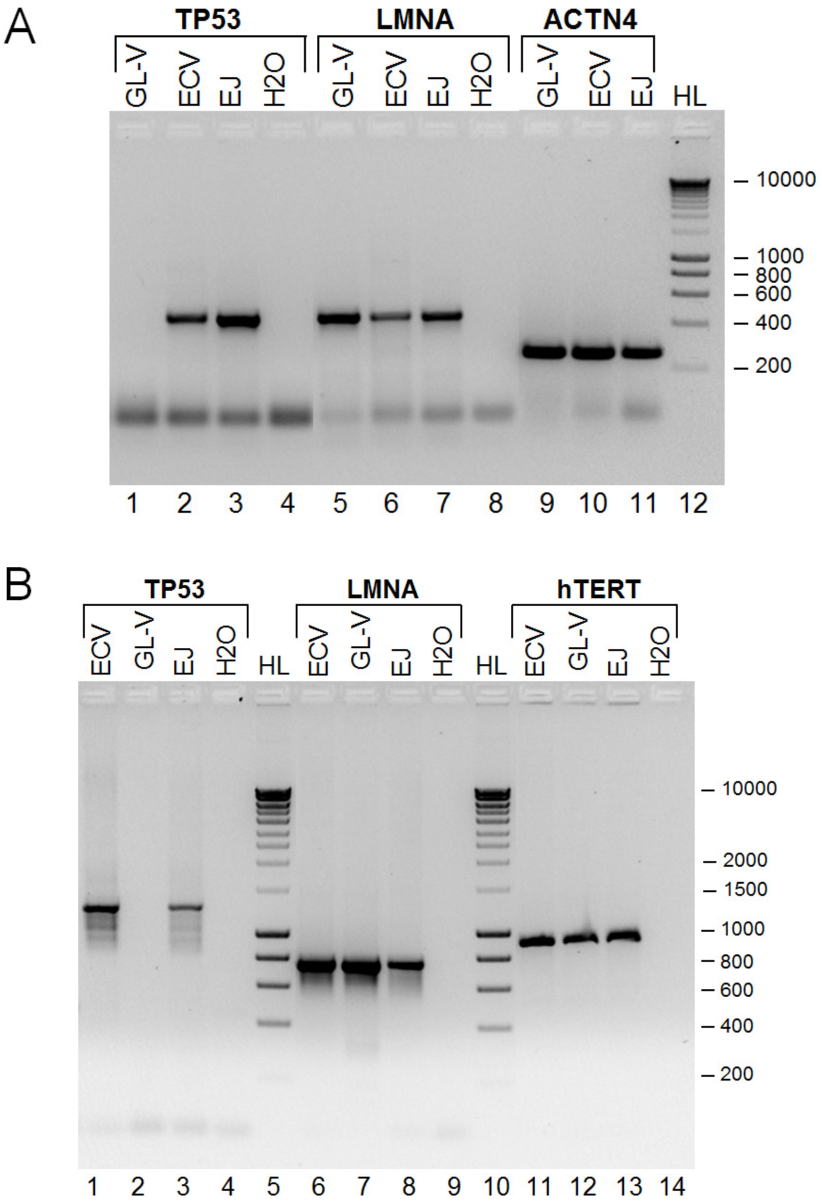

Fig S1-1. The GL-V cell line contains the deletion encompassing exon 4-5-6 of the p53 gene. (A) Agarose gel electrophoresis analysis of PCR amplification of the p53 (lane 1-4), LMNA (lane 5-8) and ACTN4 (lane 9, 10, 11) coding sequences from cDNA prepared from total RNA extracted from GL-V (lane1, 5, 9); ECV-304 (lane 2, 6, 10); EJ (lane 3, 7, 11) cells, including non-template controls (lane 4, 8). For p53, the primers, annealing to exon 4 (#87: p53ex4.f2) and to the end of exon 6 (#86: p53ex6.r1), encompass 447 base pairs

(bp) of the p53 ORF; for LMNA, the primers, annealing to the beginning of exon 10 (Ex10.F) and to exon 12 (Ex12.R), encompass 441 bp of the LMNA ORF; for ACTN4, the primers, annealing to exon 7 (Ex7.F) and to exon 9 (Ex9.R), encompass 260 bp of the ACTN4 ORF. Molecular weight markers are applied in lane 12. (B) Agarose gel electrophoresis analysis of PCR amplification of the p53 (lane 1-4), LMNA (lane 6-9) and hTERT genomic sequences from total DNA extracted from GL-V (lane 2, 7, 12); ECV-304 (lane 1, 6, 11); EJ (lane 3, 8, 13) cells, including non-template controls (lane 4, 9, 14). For p53, the same primers as in Part A encompass 1285 bp of the p53 gene; for LMNA, the primers, annealing to exon 9 (Ex9.F) and to intron 10 (In10.R), encompass 775 bp of the LMNA gene; for hTERT, the primers, annealing to exon 5 (Ex5.F) and to intron 6 (In6.R), encompass 917 bp of ACTN4 gene. Molecular weight markers are applied in lanes 5 and 10. The sizes of molecular weight markers from HyperLadder I (Bioline) are shown in bp on the right.

Mapping of the p53 deletion in GL-V cell line

The p53 exons and introns are annotated according to TP53-201 ENST00000269305.8 from the Ensembl database (http://www.ensembl.org/). The sizes of exons and introns are summarised in the Table 1. The exon locations in the p53 ORF are schematically shown in Fig S1-2A as well as the primers used to amplify the p53 cDNA or genomic (gDNA) fragments.

Exon Number	Intron Number	Size in nucleotides
2		74 from the start codon
	2-3	117
3		22
	3-4	109
4		279
	4-5	757
5		184
	5-6	81
6		113
	6-7	568
7		110
	7-8	343
8		137
	8-9	92
9		74
	9-10	2819
10		107
	10-11	918
11		82 to the end of ORF

Table S1. The sizes of the exons and introns within the p53 ORF.

We designed the primers to amplify either the individual exons or a combination of several exons within the p53 ORF from cDNA and genomic DNA. Each exon was successfully amplified by at least two pairs of primers. Non-template controls were always included and if there are bands in these controls the experiments were repeated using new reagents. Non-template controls are not included in the figure. Fig S1-2 shows the selected results of the exon amplification which was successful for the cDNA and gDNA prepared from human fibroblasts (NB1-T) carrying the wild type p53 gene, but not from GL-V cells. These results allowed us to conclude that the entire p53 ORF is deleted in the GL-V cell line.

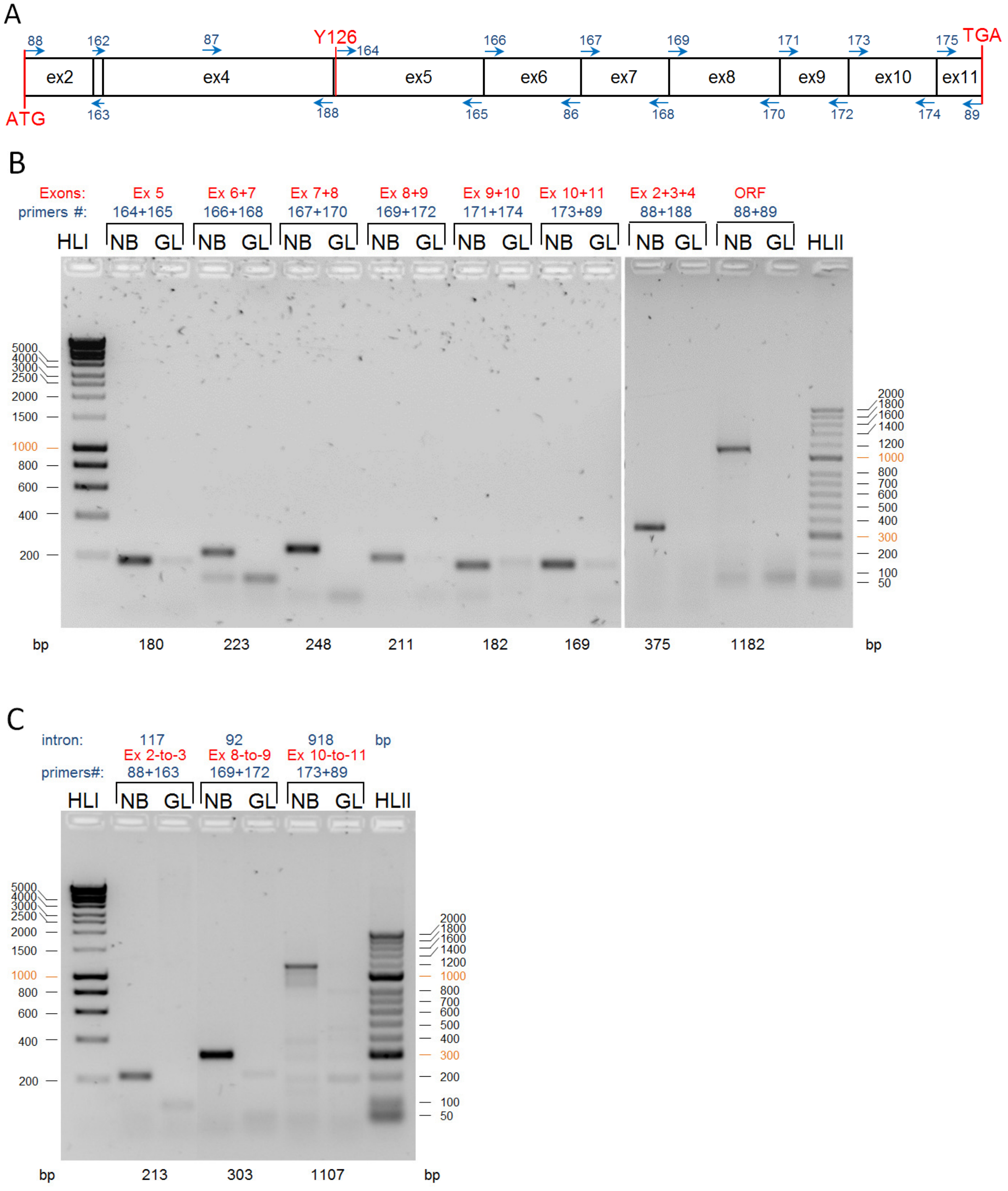

Fig S1-2. Amplification of the p53 exonic sequences from cDNA and gDNA isolated from GL-V and NB1-T cells. (A) Schematic representation of exons within the p53 ORF and the location of the primers used for PCR (the nucleotide sequences are in the list below) (B), (C) Agarose gel electrophoresis analysis of PCR products generated from cDNA and gDNA, respectively. NB and GL indicate NB1-T and GL-V cells, respectively. The primer numbers are highlighted in blue; the amplified exons indicated in red. The theoretical sizes of PCR products are indicated in bp at the bottom of the gel. The sizes of molecular weight markers from HyperLadder I and HyperLadder II (Bioline) are shown in bp on the left and on the right, respectively.

Localization of the 3' end of the p53 deletion in the GL-V cell line in the vicinity of the stop codon

To investigate whether the 3' end of the p53 deletion in the GL-V cell line could be located in the vicinity of the stop codon, we additionally designed the primers that anneal to the 3'UTR and carried out PCR using the GL-V gemonic DNA and cDNA. Fig S1-3 shows that in the presence of the primers encompassing exon 11 and the beginning of 3'-UTR (primers #175 and #205) the DNA fragments of expected molecular weight were amplified from both the GL-V gemonic DNA and cDNA, whereas the primers encompassing exon 10 and the beginning of 3'-UTR (primers #173 and #205) were unable to produce any bands. This result allowed us to conclude that the 3' end of the deletion is located between the beginning of exon 10 and the beginning of exon 11 of the p53 gene in the GL-V cell line.

A

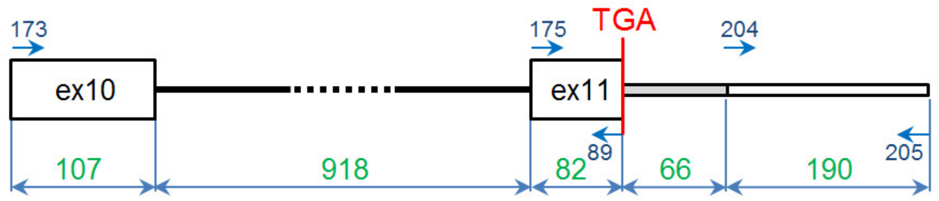

B

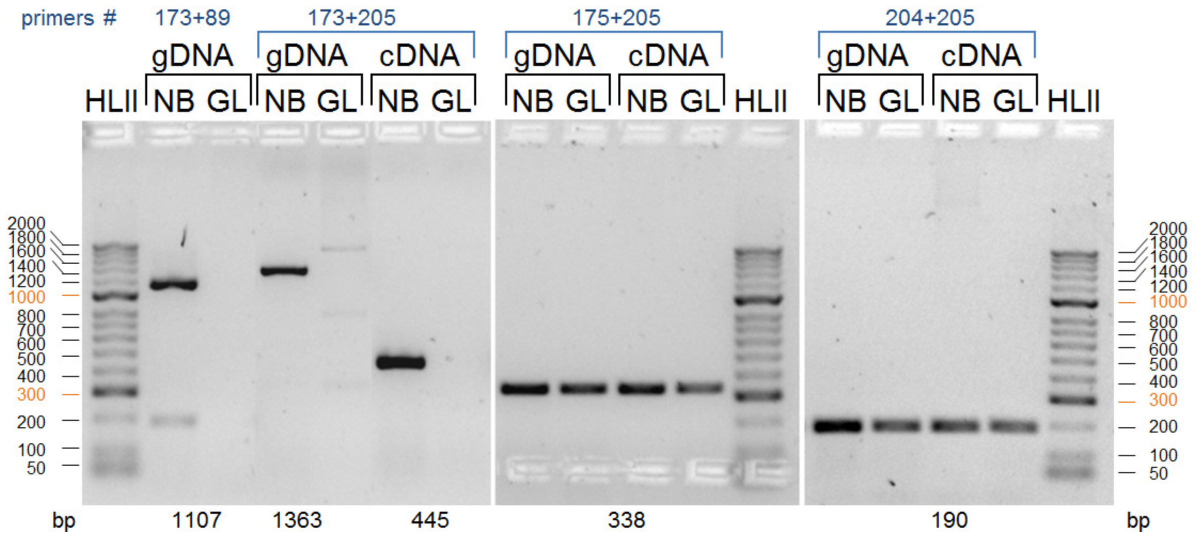

Fig S1-3. Amplification of the fragments surrounding the p53 stop codon from cDNA and gDNA isolated from GL-V and NB1-T cells.

(A) Schematic representation of the p53 genomic region surrounding the stop codon and the location of the primers used for PCR. The distances between the primers in bp are indicated in green. (B) Agarose gel electrophoresis analysis of PCR products generated from cDNA and gDNA. NB and GL indicate the NB1-T and GL-V cells. The primer numbers are highlighted in blue. The theoretical sizes of PCR products are indicated in bp at the bottom of the gel. The sizes of molecular weight markers from HyperLadder II (Bioline) are shown in bp both on the left and on the right.

List of the primers:

The p53 exon-specific primers:

#86 = p53ex6.r1:	CTCAGGCGGCTCATAGGGC
#87 = p53ex4.f2:	GCACCAGCAGCTCCTACACC
#88 = ORFp53.f1:	ATGGAGGAGCCGCAGTCAGATC
#89 = ORFp53.r1:	TCAGTCTGAGTCAGGCCCTTC
#162 = p53ex3.f1:	ACTTCCTGAAAACAACGTTCTG
#163 = p53ex3.r1:	CAGAACGTTGTTTTCAGGAAGT
#164 = p53ex5.f1:	CCCCTGCCCTCAACAAGATG
#165 = p53ex5.r1:	CATCGCTATCTGAGCAGCGCTC
#166 = p53ex6.f1:	GTCTGGCCCCTCCTCAGC
#167 = p53ex7.f1	GTTGGCTCTGACTGTACCACC
#168 = p53ex7.r1:	CTGGAGTCTTCCAGTGTGATGATG
#169 = p53ex8.f1:	GGTAATCTACTGGGACGGAACAG
#170 = p53ex8.r1:	CTCGCTTAGTGCTCCCTGG
#171 = p53ex9.f1:	CACTGCCCAACAACACCAGC
#172 = p53ex9.r1:	CTGAAGGGTGAAATATTCTCCATC
#173 = p53ex10.f1:	ATCCGTGGGCGTGAGCG
#174 = p53ex10.r1:	CTGGAGTGAGCCCTGCTC
#175 = p53ex11.f1:	CCACCTGAAGTCCAAAAAGGGTC
#188 = p53-Ex4.r2:	CGTGCAAGTCACAGACTTGGC

The p53 3'UTR-specific primers:

#204 = p53-3'UTR.f:	GGTTTTGGGTCTTTGAACCC
#205 = p53-3'UTR.r:	TCTCCCAAACATCCCTCACA

The gene-specific primers for endogenous controls:

LMNA:
Ex10.F: GAA GTG GCC ATG CGC AAG CTG; Ex12.R: GGTGAGGAG GACGCAGGAAG;
Ex9.F: GCTGCAGGAGCTGGGGC; In10.R: CCAGGCCAGCGAGTAAAGTTC

ACTN4:
Ex7.F: CGACCCTGTCACCAACCTGAAC; Ex9.R: GGCCAGCTTCTCGTAGTCCTCC

hTERT:
Ex5.F: GCGGTACCTCGAGGGTGAAGGCACTG; In6.R: GCATTCTAGACACATTCATATCCC
