# Supplementary material for: The rare nonsense mutation in p53 triggers alternative splicing to produce a protein capable of inducing apoptosis
Source: PLoS One. 2017 Sep 29;12(9):e0185126. doi: 10.1371/journal.pone.0185126 (PMC5621691; doi:10.1371/journal.pone.0185126)
Supplement: S2 File — (PDF) [file pone.0185126.s002.pdf]

Supporting Information – S2 File

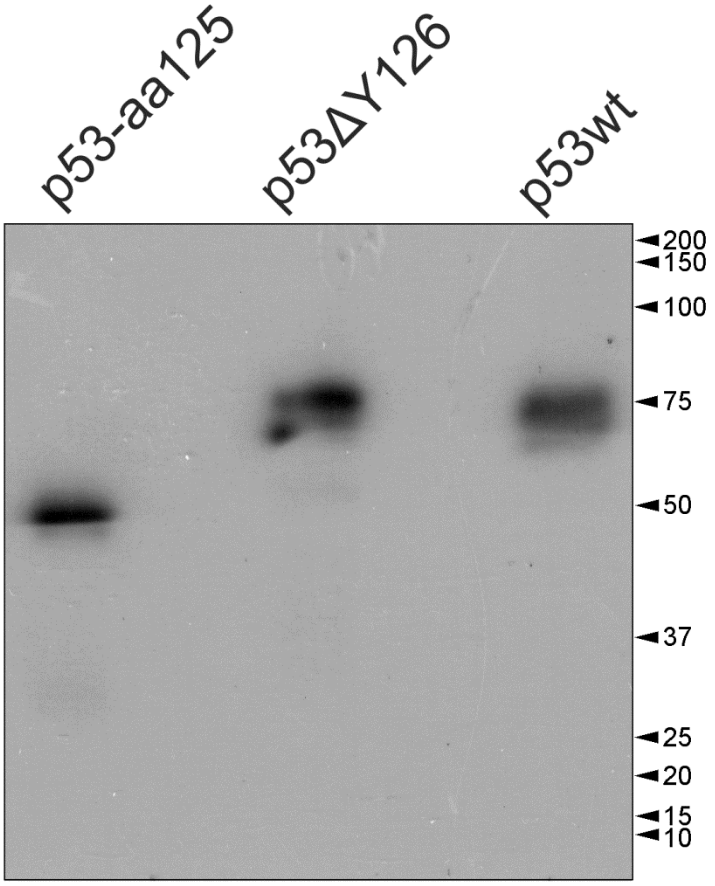

S2 Fig. Western blotting analysis of p53 expression in the K562 cells transfected with plasmids encoding the p53-aa125-EGFP, p53∆Y126-EGFP and p53wt-EGFP proteins. The K562 cells transfected with plasmids driving the expression of the different variants of p53 proteins were collected 24 hours after transfection, and the total cell extracts were analysed by Western blotting with the p53 antibody. Precision Plus Protein Dual Color standards (Bio-Rad) are indicated on the right. Note that the predicted molecular weight of EGFP is 27 kDa.
