# Supplementary material for: The rare nonsense mutation in p53 triggers alternative splicing to produce a protein capable of inducing apoptosis
Source: PLoS One. 2017 Sep 29;12(9):e0185126. doi: 10.1371/journal.pone.0185126 (PMC5621691; doi:10.1371/journal.pone.0185126)
Supplement: S3 File — (PDF) [file pone.0185126.s003.pdf]

Supporting Information – S3 File

PR00386 Motif 1 width=27

Element	Seqn Id
SGTAKSVTCTYSPSLNKLFCQLAKTCP	O36006
SGTAKSVTCTYSPCLNKLFCQLAKTCP	P53_RABIT
SGTAKSVTCTYSPDLNKMFCQLAKTCP	P53_CERAE
SGTAKSVTCTYSPDLNKMFCQLAKTCP	P53_MACFA
SGTAKSVTCTYSPDLNKMFCQLAKTCP	P53_MACMU
SGTAKSVTWTYSPLLNKLFCQLAKTCP	P53_CANFA
SGTAKSVTCTYSPPLNKLFCQLAKTCP	P53_FELCA
SGTAKSVTCTYSPALNKMFCQLAKTCP	P53_HUMAN
SGTAKSVTCTYSPALNKMFCQLAKTCP	Q16848
SGTAKSVMCTYSISLNKLFCQLAKTCP	P53_RAT
SGTAKSVMCTYSPPLNKLFCQLAKTCP	O70366
SGTAKSVMCTYSPPLNKLFCQLAKTCP	P53_MOUSE
SGTAKSVTCTYSPSLNKLFCQLAKTCP	P53_CRIGR
SGTAKSVTCTYSPSLNKLFCQLAKTCP	P53_MESAU
SGTAKSVTCTYSPSLNKLFCQLAKTCP	P53_SHEEP
SGTAKSVTCTYSPSLNKLFCQLAKTCP	P53_BOVIN
SGTAKSVTCTYSPDLNKLFCQLAKTCP	P53_BRARE
NGTAKSVTCTYSPELNKLFCQLAKTCP	P53_XENLA
SSTAKSVTCTYSPDLNKLFCQLAKTCP	P53_SALIR
AGTAKSVTCTYSPVLNKVYCRLAKPCP	P53_CHICK
SSTAKSATWTYSTELKKLYCQIAKTCP	O75080
SSTAKSATWTYSTELKKLYCQIAKTCP	O75195
SSTAKSATWTYSTELKKLYCQIAKTCP	O75922
SSTAKSATWTYSTELKKLYCQIAKTCP	O76078
SSTAKSATWTYSTELKKLYCQIAKTCP	O88897
SSTAKSATWTYSTELKKLYCQIAKTCP	O88898
SSTAKSATWTYSTELKKLYCQIAKTCP	O88899
SSTAKSATWTYSTELKKLYCQIAKTCP	O89097
SSGTKSVTCTYSPDLNKLFCQLAKTCP	O93379
SSTAKSATWTYSPLLKKLYCQIAKTCP	O15350
SSTAKSATWTYSPLLKKLYCQIAKTCP	O15351
SGTAKSVTSTYSVKLGKLFCQLAKTTP	Q92143
SGTAKSVTSTYSVKLGKLFCQLAKTTP	O57538
SGTAKSVTSTYSETLNKLYCQLAKTSP	P53_ORYLA
SGTAKSVTSTFSELLKKLYCQLAKTSP	P53_PLAFE
SKETKSTTWTYSEKLDKLYVRMATTCP	Q27937

S3 Fig. The GenBank alignment of the p53 sequences from different species surrounding the tyrosine at amino acid position 126. Y126 is highlighted in yellow; the protein accession numbers are in blue.
